# Electronic properties of amino acid side chains: quantum mechanics calculation of substituent effects

**DOI:** 10.1186/1472-6769-5-2

**Published:** 2005-08-03

**Authors:** Donard S Dwyer

**Affiliations:** 1 LSU Health Sciences Center, School of Medicine, Shreveport, LA 71130 USA

## Abstract

**Background:**

Electronic properties of amino acid side chains such as inductive and field effects have not been characterized in any detail. Quantum mechanics (QM) calculations and fundamental equations that account for substituent effects may provide insight into these important properties. PM3 analysis of electron distribution and polarizability was used to derive quantitative scales that describe steric factors, inductive effects, resonance effects, and field effects of amino acid side chains.

**Results:**

These studies revealed that: (1) different semiempirical QM methods yield similar results for the electronic effects of side chain groups, (2) polarizability, which reflects molecular deformability, represents steric factors in electronic terms, and (3) inductive effects contribute to the propensity of an amino acid for α-helices.

**Conclusion:**

The data provide initial characterization of the substituent effects of amino acid side chains and suggest that these properties affect electron density along the peptide backbone.

## Background

How the amino acid sequence of a protein determines its native tertiary structure is one of the most perplexing questions in biology. The formation of secondary structure (α-helices, β-strands and coils/turns) is an intermediate step in this process, although, in some cases, this may occur very late in folding just prior to consolidation of the final 3-D structure [1-4]. Hydrophobicity and steric effects are two major factors that govern protein folding [5-7]. In addition, I [8] have recently suggested that electronic properties of amino acids, including inductive effects, may contribute to the propensity for secondary structure. This possibility merits further investigation especially in view of several recent findings. First, although the hydrophobicity of an amino acid correlates with preference for β-strand and coil conformations, it does not predict tendency to form α-helices [8]. This suggests that adoption of β-strands *vs*. α-helices may be driven by different molecular forces. Second, electronic effects have provided important insights into structural preferences and have dramatically revised our thinking about the factors that impact rotation about a single bond. For example, the fact that ethane prefers the staggered conformation over the eclipsed conformation has long been ascribed to steric factors [9]. However, Pophristic and Goodman [10] demonstrated that hyperconjugative effects (electron delocalization into antibonding orbitals) rather than steric effects explain the conformational preference of ethane in support of earlier suggestions [11,12]. Finally, recent studies suggest that inductive effects are involved in helix formation/stabilization. Thus, inductive effects have been invoked to explain the enhanced stability of helical structures in collagen that contain fluoroproline substitutions [13,14] and to account for the preference of amino acids for α-helical structures [8]. Despite the emerging significance of electronic effects for conformational preference, little is known about the electronic properties of amino acid side chains. In order to address this shortcoming, I have applied computational chemistry, i.e., quantum mechanics (QM) calculations, to the characterization of the electronic effects of amino acids.

Electronic (substituent) effects of various chemical groups have been characterized in some detail and related to basic chemical properties including rotational flexibility and pKa [15-19]. Previously, I [8] presented theoretical arguments for considering amino acid side chains as substituents of the peptide backbone that affect electron densities and bond angles as a function of their electronic properties. Electronic effects were initially quantified in terms of the pKa at the amino group and localized electronic effects (e_σ_) estimated from the work of Charton [15]. However, as shown by, Taft [19,20], Chalvet *et al*. [16], Charton [15], and Topsom [18], the substituent effects that determine the pKa of a chemical group can be partitioned into more fundamental factors, which include inductive (through-bond) and field (through-space) effects, polarizability, and resonance effects. QM methods have been successfully applied to the derivation of substituent effects of certain chemical groups in substituted phenols [21,22], bicyclooctane carboxylic acids [15,18], and other substrates [23,24]. Until now, there has not been a detailed characterization of substituent constants for amino acid side chains.

### Theoretical considerations

The structure and properties of a molecule are determined by its electronic configuration or charge distribution [25,26]. Moreover, the electronic properties of substituent groups affect the structure, reactivity, and rotational flexibility of the substituted host molecule. Electron delocalization, including hyperconjugation in saturated molecules such as ethane, contributes to rotational freedom in molecules [10]. Rotation about the main chain bonds of proteins ultimately determines the secondary and tertiary structure of a protein, as observed by Ramachandran and colleagues [27]. It is worth noting that there is electron delocalization along the main chain, which modulates the chemical properties of proteins [28-30]. Elsewhere, I [8] have suggested that amino acid side chains can be considered substituent groups along the peptide backbone that affect the local electron distribution and rotational flexibility. However, the substituent effects of amino acids have not been systematically characterized. Therefore, a major goal of this work is to provide an initial characterization of the substituent effects of amino acids determined from QM calculations and equations that describe proton dissociation. Hammett [31] sought to account for substituent effects of chemical groups with two terms: (1) the substituent constant, σ, (reflecting intrinsic physicochemical properties of a group), and (2) a reaction constant, ρ, which specifies the nature of the reaction, the medium and temperature. Considerable evidence supports the notion that substituent effects reflect the intrinsic electronic properties of a chemical group and its environment, including temperature and solvent [15-19]. A similar concept might be applied to protein folding, i.e., the native structure is determined by inherent physicochemical properties of amino acids in concert with temperature and solvent effects. This paper lays out a general strategy for determining the inherent electronic properties of amino acid side chains, and presents an initial quantitative analysis of substituent effects that include inductive, resonance, field, and steric effects. The possible relationship of these properties to secondary structural preferences has also been explored.

## Results and discussion

### Calculation of electronic effects

Previous analysis of Hammett constants has revealed that substituent effects represent an amalgam of electronic effects. The work of Taft [20], Charton [15], Chalvet *et al*. [16], and Topsom [18] provided the theoretical framework for partitioning the substituent effects of amino acid side chains into fundamental electronic properties. The collective contribution of various factors to the electronic properties of molecules, including proton dissociation at the amino group of amino acids, can be written as:

pKa (amino group) = σ_F _+ [σ_I _+ σ_R_] + σ_α _(1)

where field effects (σ_F_), together with inductive (σ_I_) and resonance effects (σ_R_), constitute the localized electronic effect of Charton (σ*), and σ_α _represents polarizability (steric effects). The general inductive term, [σ_I+R_], consists of both inductive (σ) effects and resonance (π) effects. Inductive *versus *resonance effects can be distinguished by examination of substituent effects in saturated *versus *non-saturated ring systems such as substituted bicyclooctane carboxylic acids [15,18]. This general strategy was applied here to the characterization of a series of cyclohexanols and phenols with amino acid side chains substituted in the 4-position. The Mulliken population from QM calculations for the hydroxyl group was used as a potential indicator or reporter of electronic effects of the attached side chain groups. Changes in the electron distribution at the hydroxyl moiety mainly reflect inductive effects (sometimes equated with the electronegativity of a substituent) of the side chains in cyclohexanol and inductive plus resonance effects in phenol. Detailed derivation of inductive, resonance, and polarizability (steric) effects is described below. With this information and knowledge of amino acid pKa's, it was possible to calculate field effects with equation (1). The pKa at the amino group was used in this analysis because previous work showed a close association between electron density at this group (as measured in NMR studies) and secondary structure [32,33].

Several predictions follow from this theoretical background. First, electronic features derived from the QM calculations should be consistent across methodologies, at least for different semiempirical QM methods. Second, the electronic properties obtained from QM calculations should correlate with empirically derived substituent constants (e.g., from Charton's work). Finally, if a particular electronic effect contributes to protein folding, there should be an association between that effect and folding preference.

### Evaluation of QM methods

**Figure 1:**
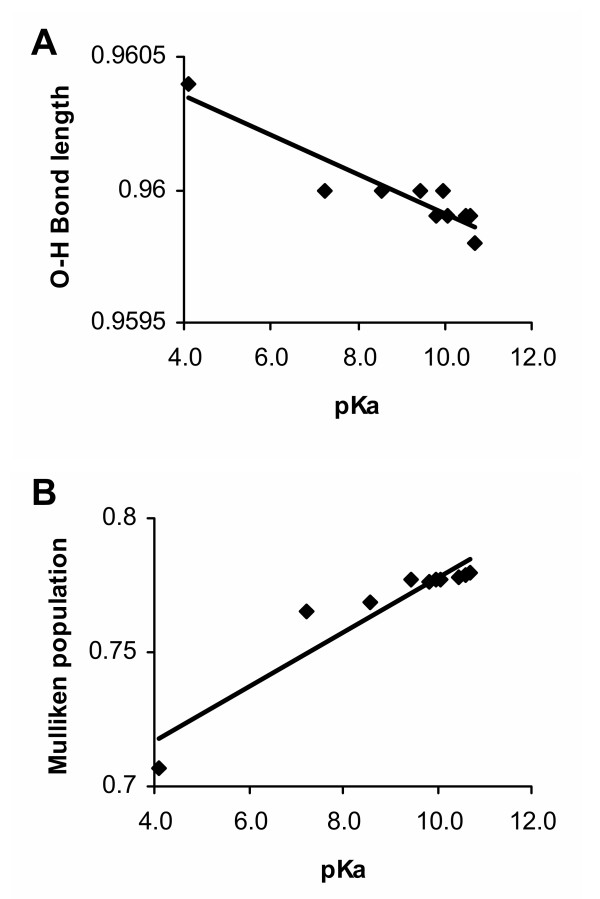
Linear regression analysis was performed to determine the correlation between experimentally-derived pKa's for a series of substituted phenols and (A) O-H bond lengths, and (B) Mulliken populations derived from PM3 calculations. The pKa values were determined by Hanai *et al*. [57].

For the QM calculations, semiempirical methods, PM3, AM1, and MNDO, were used to characterize amino acid side chains. *Ab initio *methods (both Hartree-Fock and DFT) could theoretically be employed for this analysis and may ultimately offer a more accurate picture of electronic properties of amino acids. Nevertheless, semiempirical methods are still commonly used and perform comparably to *ab initio *methods in many cases [34-37]. Thus, PM3 and MNDO QM methods were evaluated for their ability to accurately represent the electronic properties of a series of substituted phenol molecules. There was generally a good correspondence between these two semiempirical methods in the Mulliken population data calculated for the hydroxyl atoms with *r *values from linear regression analysis > 0.9 (Pearson coefficient). The PM3 method was somewhat superior overall and was chosen as the main approach for this analysis. The reliability of the PM3 calculations was established by assessing the ability of this method to predict the pKa's of a series of substituted phenols in relation to experimental data. Electron populations and bond lengths were computed for a series of phenols with substitutions (mainly at the 4-position) that included chlorine atoms, and nitro, amine and ethyl groups. Linear regression analysis revealed that there was a highly significant correlation (correlation coefficient, *r *= -0.9) between the O-H bond lengths of the substituted phenols and their experimentally determined pKa (Figure 1A). The Mulliken population data at the hydrogen atom also showed a similar high degree of correlation (*r *= 0.9) with the pKa (Figure 1B). Thus, PM3 QM values faithfully predict the dissociation behavior of the alcohol moiety in this model system.

### Quantification of the electronic properties of amino acids

PM3 calculations were then performed on each of the 20 amino acids for an initial characterization of their electronic properties. Mulliken population analysis of the heavy chain atoms and the polarizability of each residue are summarized in Table 1. There were sizeable differences among the amino acids in the Mulliken population data especially at the nitrogen and C_α _atoms. In addition, there was a significant correlation between the Mulliken population data at the nitrogen atom and the pKa at the amino group (*r *= 0.6, p < 0.01). The correlation was quite striking when cysteine was omitted from the analysis due to the anomalous pKa for its amino group. In this case, the correlation coefficient between the pKa at the amino group and the Mulliken population at the nitrogen atom was 0.8 (p < 0.005). These observations were consistent with the success of PM3 in predicting the pKa of substituted phenols on the basis of the Mulliken population data at the hydroxyl group. Mulliken values at the nitrogen atom also showed a highly significant correlation with the localized electronic effect scale of Charton (e_σ_) as compiled previously [8] (*r *= -0.7, p < 0.002). The localized electronic effect includes field, inductive, and resonance effects [8]. Therefore, the QM values calculated for the amino acids reflect complex electronic factors (pKa and e_σ_) that may be further partitioned into more fundamental components.

**Table 1:** PM3 data for the 20 amino acids.

	**Mulliken population^a^**							**Polarizability**	
	**Amino Acid**				**Cyclohexanol**		**Phenol**		**(A^3^)**

	**N**	**Cα**	**C**	**O**	**O**	**H**	**O**	**H**	
P	5.1340	4.2388	3.5314	6.6336	6.3268	0.7883	6.2674	0.7784	4.3
C	4.1918	4.6375	3.4483	6.5367	6.3264	0.7882	6.2595	0.7773	2.7
A	4.2048	4.5978	3.4552	6.5453	6.3277	0.7888	6.2615	0.7781	1.1
I	4.4251	4.4995	3.5018	6.5272	6.3281	0.7889	6.2614	0.7782	4.3
E	4.4723	4.4447	3.5051	6.6012	6.3454	0.7951	6.2811	0.7899	4.1
V	4.2068	4.6039	3.4648	6.5416	6.3267	0.7884	6.2673	0.7784	3.2
L	4.2043	4.5929	3.4581	6.5472	6.3269	0.7885	6.2609	0.7784	4.2
D	4.5761	4.3934	3.5257	6.5880	6.3482	0.7934	6.2775	0.7954	3.0
**G**	**4.1766**	**4.7053**	**3.4629**	**6.5511**	**6.3271**	**0.7883**	**6.2614**	**0.7774**	**0.03**
W	4.2111	4.5755	3.4645	6.5537	6.3272	0.7889	6.2685	0.7789	12.1
M	4.1951	4.6201	3.4468	6.5369	6.3271	0.7891	6.2620	0.7770	5.1
H	4.2906	4.5323	3.4741	6.5614	6.3285	0.7882	6.2617	0.7795	6.3
S	4.1828	4.6620	3.4635	6.5441	6.3271	0.7880	6.2617	0.7776	1.6
F	4.2128	4.5783	3.4638	6.5490	6.3272	0.7887	6.2618	0.7780	8.0
Q	4.2029	4.6050	3.4588	6.5447	6.3263	0.7873	6.2596	0.7767	4.8
Y	4.2091	4.5836	3.4611	6.5514	6.3268	0.7888	6.2620	0.7776	8.8
T	4.1873	4.6438	3.4584	6.5433	6.3265	0.7878	6.2660	0.7771	2.7
R	4.2113	4.5381	3.4861	6.5801	6.3127	0.7857	6.2469	0.7699	8.5
K	4.2248	4.5119	3.5041	6.5645	6.3137	0.7867	6.2541	0.7653	5.2
N	4.3075	4.5431	3.4674	6.5528	6.3267	0.7869	6.2608	0.7754	3.7

^a^These data refer to results of PM3 calculations for the main chain atoms of the amino acids and the hydroxyl group of cyclohexanol or phenol.

### Inductive and resonance effects of side chains

**Figure 2:**
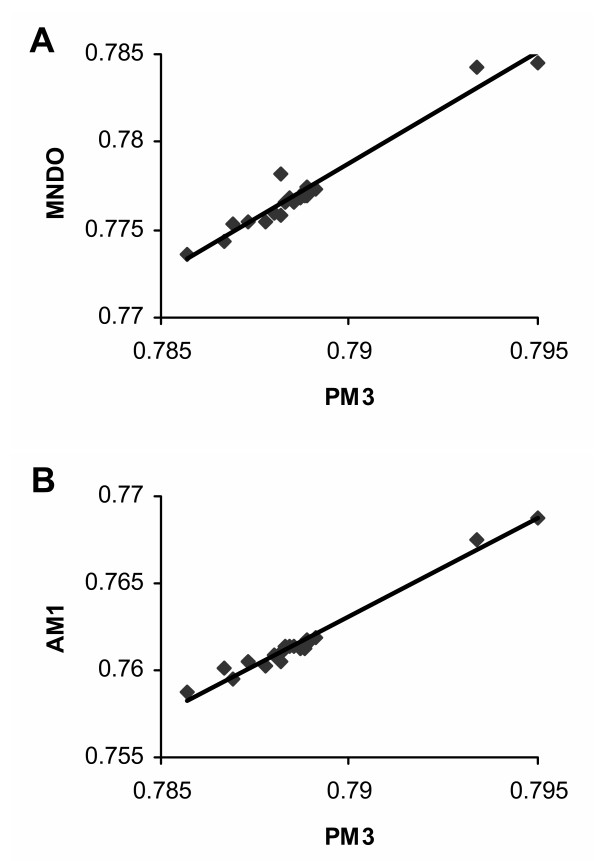
Results of linear regression analysis comparing Mulliken population data for the hydroxyl hydrogen atom in substituted cyclohexanol calculated with PM3 vs. (A) MNDO and (B) AM1 methods.

QM calculations were performed on the substituted cyclohexanol and phenol reporter molecules. The H atom (side chain) of glycine represented the zero point for the derivation of inductive and resonance effects. Values below that of glycine were assigned negative numbers to reflect the fact that decreased electron density at these atoms would encourage proton dissociation, thus tending to lower the pKa of the hydroxyl group. Specifically, the Mulliken population data for the H atom of the hydroxyl moiety were used to determine inductive effects because this value showed a high degree of correlation with the pKa of substituted phenols in the test panel. Thus, inductive effects (σ_I_) were calculated with equation (2) (see the Methods section) and reflected the difference from the glycine (cyclohexanol) reference data (Table 2). The general trends seemed reasonable because the acidic side chains of aspartic and glutamic acid produced opposite effects from the positive side chains of arginine and lysine, and alkyl groups were weak electron donors in this system as expected. There was an excellent correspondence between the σ_I _scale derived from QM calculations and the localized electronic effects of Charton (e_σ_) determined from experimental data (*r *= -0.9) (Table 3). As expected, the Mulliken population data were highly correlated (*r *= 0.99) with the electron densities calculated with PM3. Moreover, the values derived from PM3 calculations showed excellent correlation with those obtained with other semiempirical methods including MNDO (*r *= 0.98; Figure 2A) and AM1 (*r *= 0.99; Figure 2B). The fact that three separate QM methods yielded similar overall results lends support for the trends reported here even if the calculated values include a measure of uncertainty. These observations suggest that Mulliken population data can reveal fundamental behavior of a molecule in terms of electronic effects, despite potential limitations of this measure.

**Table 2:** Electronic properties of amino acid side chains.

	**pKa^a^**	**σ_I_**	**H_M_ΔPH**	**σ_R_**	**σ_α_**	**σ_F_**	**A_I_**	**HN_NMR_^b^**
P	10.60	0	0.10	0.10	-0.04	0.02^c^	0	-
C	10.28	-0.01	-0.01	0.01	-0.03	0.06^c^	0.01	8.18
A	9.69	0.05	0.05	0	-0.01	0.05	0.05	8.12
I	9.68	0.06	0.08	0.02	-0.04	0.04	0.06	7.99
E	9.67	0.68	1.25	0.57	-0.04	-1.14	0.68	8.40
V	9.62	0.01	0.09	0.08	-0.03	-0.04	0.01	8.08
L	9.60	0.02	0.07	0.05	-0.04	-0.03	0.02	7.99
D	9.60	0.51	1.80	1.29	-0.03	-1.77	0.51	8.38
G	9.60	0	0	0	0	0	0	8.36
W	9.39	0.06	0.15	0.09	-0.12	-0.24	0.06	8.03
M	9.21	0.08	-0.04	-0.12	-0.05	-0.30	0.08	8.12
H	9.17	-0.01	0.21	0.22	-0.06	-0.58	0.01	8.36
S	9.15	-0.03	-0.05	-0.02	-0.02	-0.38	0.03	8.30
F	9.13	0.04	0.06	0.02	-0.08	-0.45	0.04	7.93
Q	9.13	-0.10	-0.07	0.03	-0.05	-0.35	0.10	8.19
Y	9.11	0.05	0.02	-0.03	-0.09	-0.42	0.05	8.10
T	9.10	-0.05	-0.03	0.02	-0.03	-0.44	0.05	8.17
R	9.04	-0.26	-0.75	-0.49	-0.08	0.27	0.26	8.23
K	8.95	-0.16	-1.11	-0.95	-0.05	0.51	0.16	8.29
N	8.80	-0.14	-0.20	-0.06	-0.04	-0.56	0.24	8.33

^a^The pKa data were taken from Edsall [58]. ^b^The HN_NMR _data were derived from the NMR work of Wishart *et al*. [32] and represent chemical shift values in p.p.m. for the amide proton of residues in the coil conformation. The other terms have been defined in the text. ^c^Estimated on the basis of chemically similar groups due to anomalous pKa.

**Table 3:** Correlations between electronic properties and folding preferences^a^.

**Index**	**HN_NMR_**	**σ_I_**	**σ_R_**	**σ_α_**	**σ_F_**	**A_I_**	**C_αMULL_**
Kyte-Doolittle	-0.8**	0	0	-0.2	0.3	-0.5*	0.4
Water vapor	0.8**	0.2	0.2	0.1	-0.6*	0.5*	-0.4
Bulk	-0.3	-0.1	-0.2	0.9**	0	0.2	-0.4
Gyration	-0.2	0	-0.2	0.9**	0	0.3	-0.5*
α-helix	-0.1	0.3	0.1	0.2	-0.2	0.6**	-0.7**
β-strand	-0.8**	-0.2	-0.1	0.3	0.3	-0.4	0.2
coil	0.7**	0.1	0.4	-0.3	-0.4	0.3	-0.3
e_σ_	-0.1	-0.9**	-0.9**	0.3	0.7**	-0.6**	0.4
pKa	-0.1	0.3	0.3	-0.3	0.1	0	0
σ_I_	0.2						
σ_R_	0.2	0.8**					
σ_α_	-0.4	-0.1	-0.2				
σ_F_	-0.4	-0.8**	-0.9**	0			
A_I_	0.5*	0.7**	0.5*	0	-0.6**		
C_αMULL_	-0.2	-0.6*	-0.4	-0.2	0.5*	-0.8**	

^a^Linear regression analysis was performed to determine possible correlations between the various electronic scales presented here. The first 2 indices of hydrophobicity were taken from Kyte and Doolittle [46], the bulk scale was from Kidera *et al*. [38], and the gyration scale is that of Levitt [39]. The secondary structure preferences and e_σ _scales were described previously [8]. The *r *values from the analysis are presented here and directions of the slope are indicated by the signs. Statistical analysis of the data revealed significant correlations: *p < 0.05, **p < 0.01.

An additional scale (A_I_) is presented in Table 2 derived from the absolute values of the σ_I _index. This scale is presented to emphasize the fact that, from the perspective of the main chain atoms, there may be little difference between strong electron donation by a side chain group (e.g. acidic moieties of aspartic acid and glutamic acid) to the amino group and strong electron withdrawal from the carboxyl group by an electron acceptor (e.g. charged side chains of lysine and arginine). This suggestion is supported by the high degree of correlation (*r *= -0.8) between the A_I _scale and the Mulliken population data at the C_α _carbon (C_αMULL_) (see Table 3).

The resonance effects scale (σ_R_) was derived according to equation (3) (see the Methods section). These values showed a high degree of correlation (*r *= 0.9) with the independently-derived resonance scale of Hansch *et al*. [19], which included 7 chemical groups that correspond to amino acid side chains. Furthermore, the σ_R _scale correlated with the e_σ _constants of Charton (*r *= -0.9), which reflect a combination of inductive, field, and resonance effects.

### Polarizability index

PM3 calculations of polarizability were obtained for each of the amino acid side chains (Table 1) and a normalized polarizability index (σ_α_) was derived (Table 2). This measure reflects both the deformability and size of a substituent. Linear regression analysis revealed that the polarizability index was very similar to scales that represent steric or bulk factors of amino acids. Thus, there was a highly significant correlation with both the composite bulk scale of Kidera *et al*. [38] (*r *= 0.9) and interestingly, the side chain gyration scale of Levitt [39] (*r *= 0.9) (Table 3), which includes implicit vibrational contributions. It is known that vibrational (Raman) spectra of peptides display consistent shifts in relation to the size of the amino acid side chain [40]. Information about the correct sign to apply to the σ_α _scale in equation (1) derives from two observations. First, others have assigned a negative value to polarizability effects on protonation [23]. Second, steric effects are known to encourage proton dissociation and lower the pKa [25].

### Field effects of amino acids

The next step was to calculate the field effects of the amino acid side chains by substituting into equation (1). Field effects include electrostatic interactions between charged side chains and main chain groups and polarization effects from H-bonding between OH and NH groups of the side chains and the peptide backbone. To solve equation (1), the various indices (σ_F_, σ_I_, etc.) were weighted equally, which represented a first approximation of the relative contributions to the pKa. Charton and others [15,16] employed weighting factors in the range of 0.5–2 for similar analyses of substituent constants, so the basic assumption in the present work was consistent with these values. Normalized indices were established for polarizability and inductive/resonance effects by multiplying raw calculations by 0.01 or 100 so that the individual components of equation (1) were on the same scale (equal weighting). The work described here was focused on the relative electronic properties of amino acid side chains and not the absolute value for field effects, inductive effects, etc. In order to simplify the calculations for this analysis, the pKa values at the amino group were referenced to glycine (0), e.g., asparagine was -0.80 (rather than 8.80) and proline was 1.0 (rather than 10.60), and an arithmetic scale was used. The field effects index (σ_F_) derived from these calculations is summarized in Table 2. The high correlation between σ_F _and the independently-derived localized electronic effect scale of Charton (e_σ_) supported the overall validity of this measure of field effects (*r *= 0.7; Table 3).

To summarize the findings thus far, it was shown that the Mulliken population data for amino acid side chains revealed similar trends when several different semiempirical methods were used for the QM calculations. Second, the Mulliken population at the nitrogen atom and the polarizability scale were highly correlated with empirical data concerning the pKa and steric effects, respectively, of amino acids. Finally, the scales for inductive, resonance, and field effects showed strong correlation with the localized electronic effect scale of Charton (e_σ_), which was derived from experimental observations.

### Relationship to secondary structure

The next objective was to determine whether any of the electronic scales correlated with the folding preferences of the amino acids. A clear relationship between a particular electronic property and secondary structure preference might provide fundamental insights into the forces that drive protein folding. Moreover, although the hydrophobicity of an amino acid is a good predictor of its preference for β-strand and coil conformations, this measure is a poor predictor of helix propensity [8]. The secondary structural preferences used for this analysis were derived previously [8] from an analysis of over 24,000 residues. Our scales show good (0.72–0.8) [41-43] to excellent (0.83–0.93) correlation [44,45] with structural preferences reported by other groups. Of the indices presented here, the empirical HN_NMR _index is the best predictor of secondary structure at least for β-strand and coil conformations (Table 3). Given the close correlation between the HN_NMR _scale and various hydrophobicity scales, this relationship is not surprising. Furthermore, the correlation between hydrophobicity and β-strand and coil preference is confirmed here for both the Kyte-Doolittle scale [46] (*r *values: coil, -0.6; β, 0.7; α, -0.1), and the partition coefficient in water vapor (coil, -0.7; β, -0.7; α, 0.06) (Table 3). However, these scales are completely inadequate for predicting the propensity of amino acids for α-helical conformations. The simple electronic property that best predicts preference for α-helices is the Mulliken population at the Cα atom (C_αMULL_) derived from the PM3 calculations (*r *= -0.7). Previous work from this laboratory suggested that electronic effects along the peptide backbone contribute to α-helical preference [8]. Although the inductive scale (σ_I_) in Table 2 does not predict the propensity of amino acids for α-helices, the absolute value of this index (A_I_) shows a significant correlation with helix preference (*r *= 0.6; Table 3). The A_I _scale is highly correlated with the C_αMULL _values (*r *= -0.8).

One possible interpretation of these findings would be that opposite processes related to electron delocalization along the peptide backbone produce similar conditions that favor formation of α-helices. More specifically, electron donation by a side chain (e.g., glutamic acid) to the amino group and electron withdrawal by a side chain (e.g., lysine) from the carboxyl group may exert similar overall effects on the electron distribution along the main chain. In both cases, the inductive effects of the side chains disrupt the normal electron flow from the carboxyl to the amide group. The net result would be a decrease in π-character along the backbone (an increase in electron density), an increase in bond length, and enhanced rotational flexibility. This flexibility may be required for adoption of α-helices.

**Figure 3:**
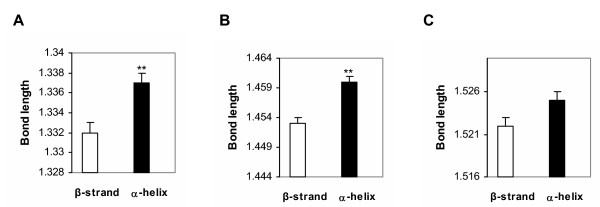
Bond lengths in high resolution structures of β-strands and α-helices. The average bond lengths (in Å) are shown for main chain bonds: (A) C-N, (B) N-C_α_, and (C) C_α_-C. In each case, the length of bonds in the α-helices is significantly longer than that in β-strands (Student's t-test, p < 0.01).

The hydrophobicity of amino acids reasonably predicts strand and coil conformations, but is a poor predictor of α-helices. Nevertheless, solvent effects clearly help to drive protein folding. In contrast to hydrophobicity, electronic scales that predict α-helices (C_αMULL _and A_I_) tend to fare poorly in the prediction of other secondary structures. These observations suggest that folding into α-helices *versus *coils and β-strands may be driven by different forces. Inductive effects appear to play a significant role in helix formation, whereas polarity and solvent effects are the major determinants of other secondary structures. Thus, helix formation is opposed by high polarity near the main chain and by disruption of inductive effects. Amino acids that prefer α-helices have a higher average electron density at the main chain atoms (from PM3 calculations), which would mean longer bond lengths and greater rotational freedom. By contrast, amino acids with a propensity for β-strands tend to have a lower electron density at the main chain atoms, which would produce the opposite effects. These predictions received initial support from an analysis of bond lengths in β-strands vs. α-helices in a panel of 7 proteins with high resolution (< 0.93 Å) crystal structures. As seen in Fig. 3, bonds involving the nitrogen atom along the main chain of α-helices are slightly, but significantly, longer than those of the β-strands, which is consistent with the increased electron density at this atom determined from our QM calculations and NMR data [32]. The longer bonds imply greater rotational freedom and less π character in α-helices compared to β-strands. The proposal that electron densities at the main chain atoms ultimately determine α-helix propensity is consistent with the observations of Wishart *et al*. [32] and of Creamer and Rose [7] who concluded that "general factors that drive helix formation must originate in the backbone." Furthermore, this notion is consistent with the role of inductive effects in the formation of helical structures as suggested by earlier studies [8,13,14]. Here, we have independently arrived at the critical role of inductive effects in helix formation and have for the first time provided quantitative estimates of the inductive effects of the 20 natural amino acids.

### Additional determinants of protein folding

The main goal of these studies was to provide a more precise description of the electronic properties of amino acids in order to relate these features to protein folding. Few studies have explored this topic despite the fact that a better understanding of folding hinges on a detailed analysis of electron distributions and molecular orbitals of the main chain atoms. Towards this end, electronic properties of amino acid side chains have been derived from two major sources: quantum mechanical (PM3) calculations of Mulliken populations and the solution of equations that relate substituent effects to inductive effects, field effects, and polarizability. Semiempirical QM methods have been used with success to predict electronic effects such as charge transfer [47], proton affinities [48,49], rotational states related to protonation [50], and heat of formation [34,35]. Although more recent *ab initio *methods (including the application of density functional theory) may prove superior, in some cases the results with semiempirical approaches have been comparable to those obtained with more demanding *ab initio *calculations [35-37]. QM calculations have previously been used to define electronic effects of substituents in terms of surrogate measures that include electron densities and bond lengths at proton donor groups [18,22-24,51]. For example, bond lengths in pentaoxyphosphoranes calculated from *ab initio *methods showed a highly significant correlation with experimentally measured pKa's [51]. However, the derivation of electronic properties is potentially limited by certain factors such as the relative weighting of the various scales in the solution of equation (1) and the accuracy of the QM calculations. Nevertheless, Topsom [18] concluded that absolute measures from QM calculations are not necessary for most studies of substituent effects. Hopefully, this preliminary analysis will stimulate further development of the conceptual framework needed to precisely define the electronic features of amino acid side chains.

Notwithstanding these potential limitations, the work presented here reveals a potential role of electronic factors, in particular inductive effects, in determining preference for secondary structure. The significance of these effects should not be underestimated because studies have shown that inductive effects extend across non-conjugated bonds in proteins [30] and may even affect electron density over a distance of several residues [52]. Despite progress in characterizing factors that affect protein folding, hydrophobic effects and electronic effects do not fully account for the structural preferences of amino acids. Most likely, the remaining forces that contribute to folding result from two types of context effect: nearest neighbor and tertiary stabilization effects [4]. Tertiary stabilization refers to the observations of Kabsch and Sander [53] that the same 5 amino acids could be found in both α-helical and β-strand conformations in different proteins. Presumably in attaining the energy minimum of the whole protein, smaller modules may assume secondary structures that do not represent the energy minimum of that particular module owing to contact-assisted structural consolidation during condensation of folding [4]. Of course, tertiary stabilization ultimately involves various electronic effects: electrostatic interactions, dispersion forces, dipole alignment, and rotational flexibility (including hyperconjugation).

## Conclusion

This paper presents a thorough description of the electronic properties of amino acid side chains. Quantitative scales were derived for representing inductive, resonance, and field effects, and polarizability (steric) factors. Regression analysis revealed that Mulliken population values at the C_α _atom and inductive effects were the best predictors of helix preference. Thus, preference for secondary conformation appears to be influenced by the electronic properties of amino acid side chains. With further refinement of these properties, it may be possible to describe protein folding purely in electronic terms, including electron densities, inductive effects, field effects, and polarizability. The correlation data presented here suggest that such a strategy may yield important new insights into factors that promote the folding of proteins.

## Methods

### QM calculations

Computational analysis was performed with a Silicon Graphics Indigo^2 ^workstation outfitted with the Insight II software package (Accelrys; San Diego, CA). PM3 [54] calculations of Mulliken populations and polarizability were performed using the MOPAC program with restricted Hartree-Fock methods. For comparison, MNDO [55] and AM1 [56] methods were also used to calculate properties in initial studies. The electronic features of amino acids were analyzed in one of several contexts. With the exception of proline, individual amino acids were evaluated in their zwitterion form in order to gain insight into their electronic properties independent of the context of a protein. Analysis of the various molecules was performed in the absence of solvent to simplify the system and to focus on inherent tendencies of amino acid side chain groups. The side chains of aspartic acid and glutamic acid carried a net charge of -1 e.u., whereas the side chains of arginine and lysine were +1 e.u. All other side chains were neutral.

In order to distinguish inductive *vs*. resonance effects, in some QM calculations the amino acid side chains (from C_β _outward) were attached at the 4-position to the reporter molecules cyclohexanol and phenol, which in their unsubstituted forms represented glycine (i.e., a hydrogen atom side chain). The geometries of the amino acids and substituted rings were optimized *a priori *through energy minimization and data were averaged from the two lowest energy structures. Both the absolute and relative values for the Mulliken populations for these two conformations were very consistent with a correlation coefficient of 0.99. The electronic structure of amino acids in other conformations will be somewhat different; however, analysis of a myriad of possible higher energy structures is not possible. Consequently, we have focused on the lowest energy conformations to derive intrinsic properties of amino acid side chains. Small deviations from the lowest energy conformation have little effect on the overall QM calculations (*r *= 0.99), whereas large deviations from this structure are uncommon and therefore less reflective of inherent properties. The behavior of the hydroxyl moiety (bond lengths and Mulliken populations) in substituted cyclohexanol and phenol was evaluated as an indicator of the substituent effects of the side chains. Various groups have used a similar approach to study the effects of other types of chemical substituents [18,22,23,51]. Mulliken population values derived from the zwitterion data are summarized in Table 1 for the heavy chain atoms of the 20 amino acids. In addition, polarizability values (see next section) derived from these calculations are presented.

### Derivation of the polarizability (σ_α_) scale

Charton [15] and Chalvet *et al*. [16] included steric factors in their derivation of substituent effects, whereas Topsom [18] and Graton *et al*. [23] included a polarizability term in their equations. It appears that both terms refer to the same effect, namely the overall size and deformability of a chemical group. Thus, we considered these terms to be roughly equivalent. Because polarizability can be evaluated directly from QM calculations, this is the convention that has been adopted for the present work. The average polarizability (α component) was determined with PM3 calculations as described above. The original values for the 20 amino acid side chains ranged from 1–13 Å^3^. In order to normalize the various substituent scales, these original values were multiplied by a factor of 10^-2 ^to obtain the data presented in Table 2.

### Derivation of inductive (σ_I_) and resonance (σ_R_) scales

In order to tease apart inductive *versus *resonance effects of substituents, various groups have characterized the effect of a substituent in the context of π interactions (i.e., attached to a phenol ring) and compared this with effects produced in molecules that lack significant resonance, such as bicyclooctanes or cyclohexane [15,18]. A similar approach was used here to characterize the amino acids. Side chain atoms from C_β _outward were bonded to cyclohexanol or phenol at the 4-position with the Biopolymer module of the software package. Cyclohexanol and phenol served as the standards for comparison and represented the glycine side chain. The structures were subjected to extensive energy minimization prior to QM calculations with the PM3 semiempirical method. Key values for the hydroxyl atoms provided the basis for derivation of the electronic properties of amino acid side chains. Inductive effects (σ_I_) of side chains were derived from Mulliken population analysis of the hydroxyl hydrogen atom (H_MULL_) in cyclohexanol according to equation (2), where aa represents any amino acid and gly represents the glycine reference data (cyclohexanol).

(H_MULL_aa – H_MULL_gly) X 100 = H_M_ΔCY (2)

The values were multiplied by 100 in order to normalize them in relation to the pKa values. These normalized Mulliken population data are referred to in this section as H_M_ΔCY. These values also represent the inductive effects (σ_I_) of the amino acid side chains. Similar normalized Mulliken population data for the amino acid side chains in the context of phenol (H_M_ΔPH) were calculated from the PM3 results. The resonance effect (σ_R_) scale was then derived according to equation (3).

H_M_ΔPH – H_M_ΔCY = σ_R _(3)

### Bond length analysis

A panel of 7 proteins was selected from the Protein Data Bank on the basis of their high resolution (< 0.93 Å) crystal structures and inclusion of both α-helices and β-strands. The panel included: crambin (1ejg; 0.54 Å resolution), aldose reductase (1us0; 0.66 Å), syntenin (1r6j; 0.73 Å), subtilisin (1gci; 0.78 Å), α-lytic protease (1ssx; 0.83 Å), ribonuclease (1dy5; 0.87 Å), and cholesterol oxidase (1n4w; 0.92 Å). Bond lengths along the main chain of randomly selected secondary structures were measured automatically. A total of 450 bonds were examined in α-helical conformations and 343 in β-strands.
